# Activation of Pluripotency Genes in Human Fibroblast Cells by a Novel mRNA Based Approach

**DOI:** 10.1371/journal.pone.0014397

**Published:** 2010-12-30

**Authors:** Jordan R. Plews, JianLiang Li, Mark Jones, Harry D. Moore, Chris Mason, Peter W. Andrews, Jie Na

**Affiliations:** 1 Biomedical Science Department, Centre for Stem Cell Biology, University of Sheffield, Sheffield, United Kingdom; 2 Department of Biochemical Engineering, University College London, London, United Kingdom; 3 School of Medicine, Tsinghua University, Beijing, China; Tufts University, United States of America

## Abstract

**Background:**

Several methods have been used to induce somatic cells to re-enter the pluripotent state. Viral transduction of reprogramming genes yields higher efficiency but involves random insertions of viral sequences into the human genome. Although induced pluripotent stem (iPS) cells can be obtained with the removable PiggyBac transposon system or an episomal system, both approaches still use DNA constructs so that resulting cell lines need to be thoroughly analyzed to confirm they are free of harmful genetic modification. Thus a method to change cell fate without using DNA will be very useful in regenerative medicine.

**Methodology/Principal Findings:**

In this study, we synthesized mRNAs encoding OCT4, SOX2, cMYC, KLF4 and SV40 large T (LT) and electroporated them into human fibroblast cells. Upon transfection, fibroblasts expressed these factors at levels comparable to, or higher than those in human embryonic stem (ES) cells. Ectopically expressed OCT4 localized to the cell nucleus within 4 hours after mRNA introduction. Transfecting fibroblasts with a mixture of mRNAs encoding all five factors significantly increased the expression of endogenous OCT4, NANOG, DNMT3β, REX1 and SALL4. When such transfected fibroblasts were also exposed to several small molecules (valproic acid, BIX01294 and 5′-aza-2′-deoxycytidine) and cultured in human embryonic stem cell (ES) medium they formed small aggregates positive for alkaline phosphatase activity and OCT4 protein within 30 days.

**Conclusion/Significance:**

Our results demonstrate that mRNA transfection can be a useful approach to precisely control the protein expression level and short-term expression of reprogramming factors is sufficient to activate pluripotency genes in differentiated cells.

## Introduction

An approach to reprogram cell fate without genetic modification would be very useful for regenerative medicine. Currently, most methodologies go through DNA-based routes, with foreign genetic materials either permanently left in the genome of resulting cells, or later removed or lost after multiple rounds of cell division. In all the cases, stringent genome wide tests are needed to confirm the absence of potentially harmful insertional mutagenesis [1], [2]. Protein transduction of recombinant transcription factors has been used for reprogramming [3], but proteins produced in bacteria may be mis-folded and lack essential modifications that only occur in mammalian cells, so that their in vivo functionality may be compromised. Kim and colleagues reported the generation of human iPS cells by incubating fibroblasts with lysates from HEK 293T cells expressing recombinant OCT4, SOX2, KLF4 and cMYC [4]. However, cell lysates contain many poorly defined factors that could also be taken up by the reprogrammed cells and give unpredictable consequences. Thus this method will be difficult to implement in clinical settings. While chemical compounds have been used to regulate cell fate or alter DNA and chromatin modifications, to date, no reprogramming or trans-differentiation has been achieved by small molecules alone [5].

An mRNA-based approach could offer several advantages: first, it does not lead to any genetic modification of the host genome. mRNAs are directly translated into functional proteins in the cytoplasm with proper mammalian post-translational modifications which would result in much higher functionality than recombinant proteins produced in the bacteria. Second, mRNAs are much smaller than DNAs, and as single strand nucleic acids without any flanking plasmid sequences they can be introduced into cells with higher efficiency and much lower cytotoxicity. It is also easier to combine several different mRNAs and to control their dosage than using multiple or multi-cistronic DNA constructs. An obvious disadvantage of mRNAs is that they are degraded by the cell in 2-3 days so that the expression window is very short. Nevertheless, an mRNA based approach could be a useful means to regulate cellular function, and to mediate trans-differentiation such as from fibroblast to neurons or cardiomyocytes [6], [7] that require shorter time.

mRNA transfection has been used in hematopoietic progenitor cells, mesenchymal stroma cells, antigen presenting dendritic cells and lymphocytes [8], [9], [10], [11]. Activated B cells transfected with mRNAs encoding co-stimulatory molecules, cytokines and antigen showed enhanced proliferation and were able to induce antigen-specific cytotoxic T lymphocyte responses [10]. dendritic cells transfected with mRNAs of viral antigen stimulated robust and specific T cell response [12]. Moreover, in a phase I/II clinical trial of dendritic cells vaccine, autologous dendritic cells loaded with autologous melanoma mRNA as tumor antigen produced vaccine specific response in the majority of patients [13]. This trial also showed that cells transfected with mRNA are safe for use in patients.

In the present study, we set out to test the feasibility of using mRNA to induce pluripotency. We found that microporation is highly effective for mRNA transfection. Moreover, transient expression of OCT4, SOX2, KLF4, cMYC and LT together with small molecule treatment significantly increased the expression of embryonic stem cell specific genes in fibroblast cells.

## Methods

### Ethics Statement

HuF1 (XX) was derived from an abortus obtained from a patient undergoing 1st trimester fetal termination using Mefipristone. The project was approved by the South Sheffield Research Ethics Committee (SSREC) and a fully informed patient consent (written) was obtained according to local and national guidelines.

### Cell culture

Three human fibroblast lines were used in this study. HuF1 (XX, passage 4) is a human fetal skin fibroblast cell line (source described above). The derivation procedure was as follows: fetal skin was recovered and chopped in to small pieces in DMEM medium supplemented with 15% fetal calf serum. An outgrowth of fibroblast cells proliferated to confluency and were passaged to passage 4 using trypsin-EDTA before being cryopreserved in 10% DMSO in FCS. MRC5 (ATCC, CCL-171, XY, passage 15) is a human embryonic lung fibroblast line and HFF (ATCC CRL-2429, XY, passage 4) was derived from human foreskin. Human fibroblast cells were maintained in DMEM 10% FCS, 10% CO_2_. After transfection of reprogramming factors, they were seeded on gelatin coated flasks (BD), cultured in MEF conditioned human embryonic stem cell medium (HES Medium) containing 20% Knock-out serum replacement (KSR) [14] and 8 ng/ml of FGF2. In some cases 200 µM valproic acid (Merck), 1 µM BIX01294 (Tocris) and 0.5 µM 5-aza-2′-deoxycytidine (Sigma) were included.

### Cloning and mRNA in vitro transcription

cDNAs encoding OCT4, SOX2, KLF4,cMYC were cloned from human ES cells using the One-step RT-PCR kit (Invitrogen). SV40 large T cDNA was a generous gift from Dr. Robert Weinberg. The identity of each gene was confirmed by sequencing. The coding regions were all inserted into the RN3P plasmid between a T3 RNA polymerase promoter and a recombinant polyA tail [15]. For mRNA in vitro transcription, the plasmids were linearized with Sfi I, and the capped mRNAs were synthesized using an AmpliCap-Max T3 High Yield Message Maker kit (Epicentre).

### Electroporation

Human fibroblast cells, keratinocytes and neural stem cells were electroporated using a Microporator Neon (Invitrogen), using pre-optimized parameters – herein referred to as ‘microporation’. Following microporation, cells were transferred into pre-warmed fibroblast medium (DMEM with 10% FCS). Next day, the medium was replaced with MEF conditioned HES medium supplemented with FGF2 (8 ng/ml). In some experiments, valproic acid (0.2 µM) (Merck), BIX01294 (1 µM) (Tocris) and/or 5-aza-2′-deoxycytidine (0.5 µM) (Sigma) were also added.

### Western blot

The following antibodies are used: OCT4 (Santa Cruz, sc-5279), NANOG (R&D systems, AF1997), SOX2 (Chemicon, AB5603), cMYC (Santa Cruz, sc-764), KLF4 (Santa Cruz, sc20691), LIN28 (R&D systems, AF3757). Cells were trypsinized, washed with PBS three times and lysed in sample loading buffer (0.125 M Tris-HCl, pH = 6.8, 4% SDS, 20% Glycerol, 0.002% Bromophenol Blue). 2×10^5^ cells equivalent lysate was loaded per lane.

### Reverse transcription and Q-PCR

RNA was extracted with TRIZOL (Invitrogen). Q-PCRs were carried out with SYBR Green JumpStartTM Kit on a Bio-Rad iCycler. The sequences of the primers used are listed in Table S1.

### Immunostaining and alkaline phosphatase (ALP) assay

For immunostaining, cells were fixed with 4% PFA and stained with OCT4 antibody followed by Alex488 conjugated 2^nd^ Ab. ALP assay was performed using the ALP substrate solution (Sigma AR0100 and AR0200) according to manufacturer's instruction.

## Results

### Producing mRNAs of reprogramming factors with recombinant 5′ and 3′ UTRs

**Figure 1:**
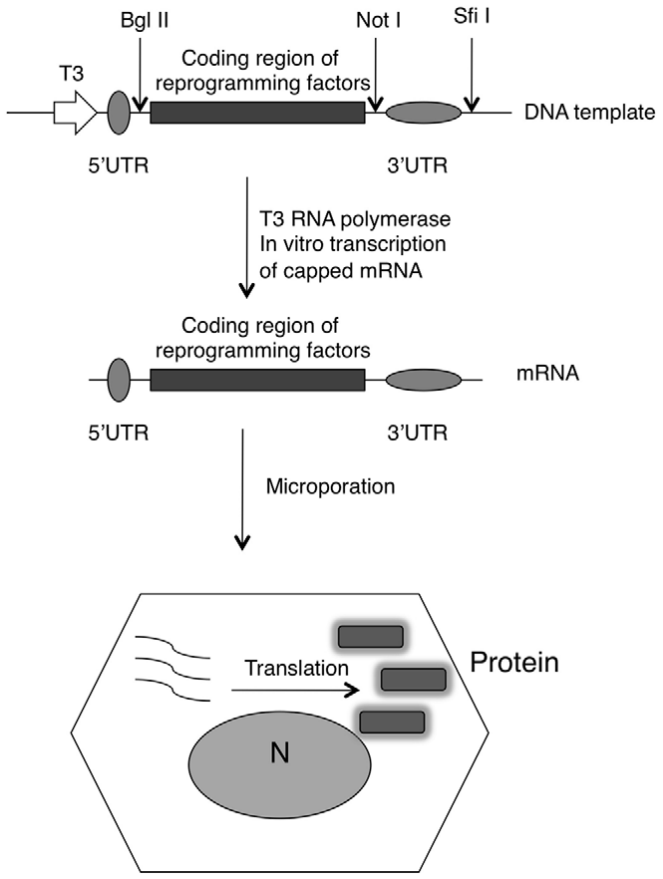
Schematic view of experiment strategy.

To generate mRNAs of reprogramming factors efficiently, we employed RN3P vector that contains the 5′ and 3′ UTRs of *Xenopus* β-globin flanking the multiple cloning sites (MCS) [15]. The 5′UTR contains the ribosomal binding site to enhance translation initiation and the 3′UTR can stabilizes the mRNA (Fig. 1). The coding sequences of OCT4, SOX2, KLF4, cMYC and SV40LT were inserted between the Bgl II and Not I sites. The plasmids were first linearized with Sfi I, and T3 RNA polymerase was used to transcribe mRNAs.

### Efficient transfection of human fibroblast cells with mRNA by microporation

**Figure 2:**
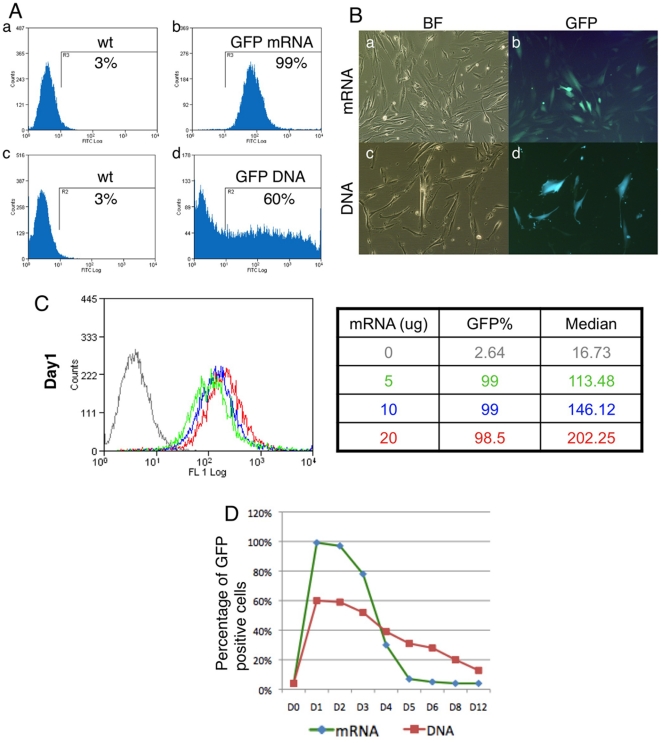
Efficient transfection of GFP mRNA into human fibroblast cells. (A) FACS histogram of GFP positive cells after GFP mRNA or DNA transfection (b and d). Non-transfected cells (a and c). (B) GFP mRNA and DNA transfected cells. BF brightfield. (C) Histogram and table of GFP positive cells after microporation of 0, 5, 10 and 20 µg of mRNA. (D) Percentage of GFP positive cells over 12 days following mRNA or DNA transfection.

To establish a method to introduce mRNA into human fibroblast cells, we utilized the Neon Transfection system (Invitrogen). We found that by optimizing electroporation parameters, we could consistently obtain nearly 100% transfection efficiency with GFP mRNA in MRC5 cells, while only 50–60% cells are positive for GFP after DNA plasmid microporation (Fig. 2A). mRNA has significant lower cytotoxicity compared to DNA, as we observed 2–3 times more cells survived after mRNA microporation than after DNA transfection (data not shown). Increasing the amount of mRNA used for transfection led to a corresponding increase in the median fluorescence intensity quantified by flow cytometry, while the percentage of the cells positive for GFP was still close to 100% (Fig. 2D). We also monitored the duration of GFP expression. More than 95% of MRC5 cells expressed GFP one and two days after microporation, whereas the percentage decreased sharply from day 3 and reached background levels by day 5. The percentage of GFP positive cells after DNA microporation was close to 60% in day 1 and 2, then decreased gradually during the following days (Fig. 2D).

**Figure 3:**
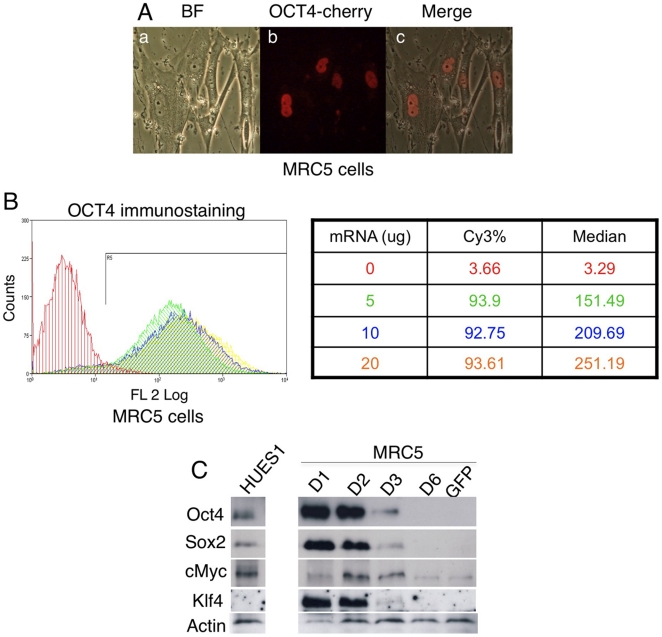
Protein expression following mRNA transfection. (A) OCT4-RFP localizes into nucleus in fibroblast cells. (B) FACS analysis of OCT4 protein expression 24 hrs after mRNA microporation. Cy3 conjugated secondary antibody was used. (C)Western blot showing corresponding protein expression in 10^6^ MRC5 cells transfected with OCT4 (17 µg), SOX2 (10 µg), cMYC (6 µg), KLF4 (6.5 µg), SV40LT (3.5 µg). The negative control is GFP mRNA transfected MRC5 cells. HUES1 is human ES cells.

To confirm that the ectopically expressed reprogramming factors correctly localize as the endogenous protein, we generated a vector encoding OCT4 fused to the fluorescent protein mCherry [16]. Four hours after mRNA transfection, we could detect most OCT4-cherry protein in the nucleus (Fig. 3A). Moreover, the immunoflurescence of ectopically expressed OCT4 co-related with the amount of mRNA transfected (Fig. 3B). It is critical that the protein levels of ectopically expressed reprogramming factors were comparable to that of the ES cells. During the first two days after mRNA transfection, higher amount of OCT4, SOX2 and KLF4 protein were detected in MRC5 cells than that in human ES cells, while cMYC proteins were similar (Fig. 3C). However, the level of ectopically expressed protein decreased significantly at day 3 and became undetectable at day 4. GFP mRNA transfected fibroblast cells had low level of cMYC, while OCT4, SOX2 and KLF4 were undetectable. Taken together, these data demonstrate that mRNA microporation has the advantage of low cytotoxicity, high efficiency and an ability to control precisely the protein expression level by varying the dosage, while the disadvantage of the approach is the short expression window with the peak expression lasting only about two days.

### Individual reprogramming factors differentially affect fibroblast survival and proliferation

**Figure 4:**
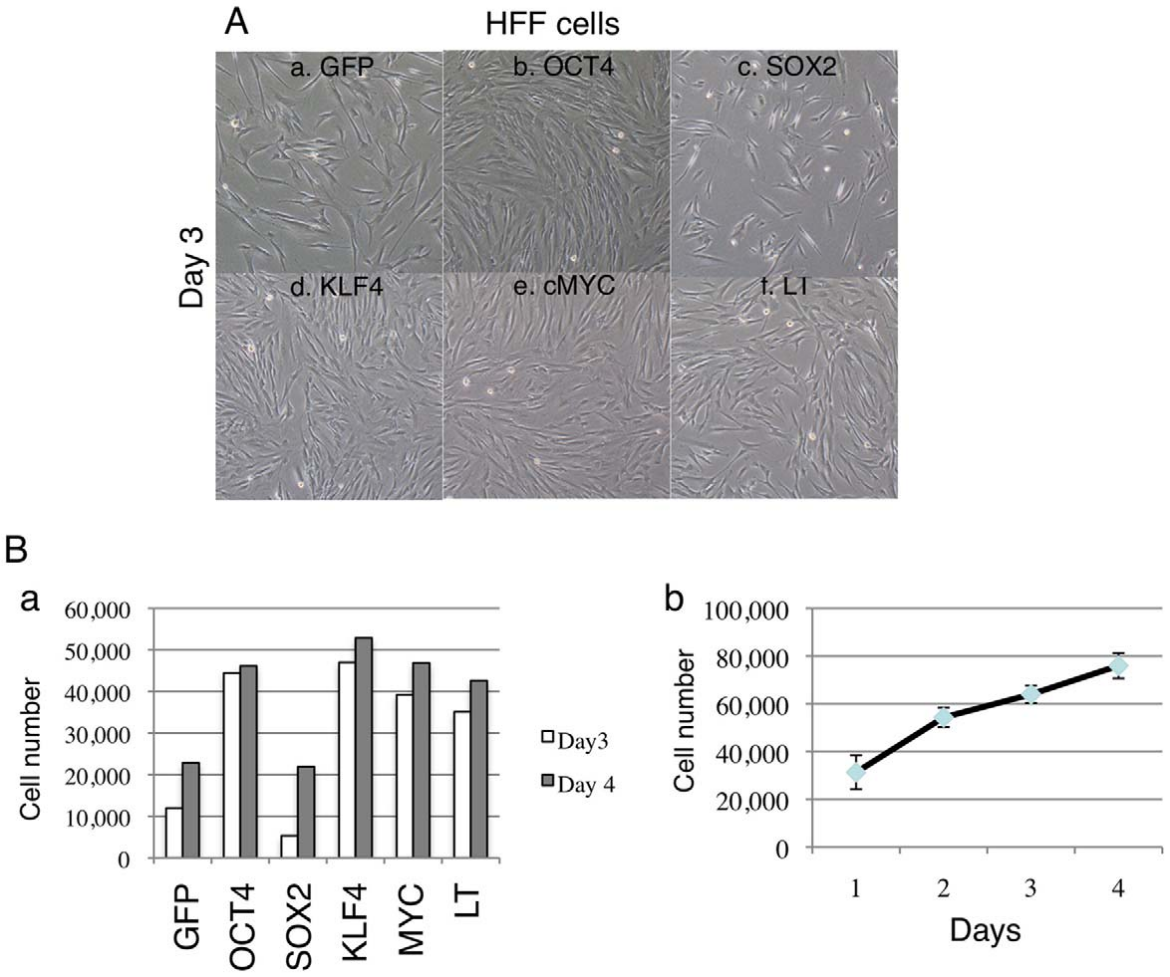
Cell survival study following mRNA transfection. (A) Morphology of cells transfected with each reprogramming factor. (B–a) Bar graph of cell number 3 and 4 days after mRNA transfection. Note that SOX2 group has much fewer cells than other groups. (B–b) Growth curve of MRC5 cells undergone microporation but no mRNA was added.

**Figure 5:**
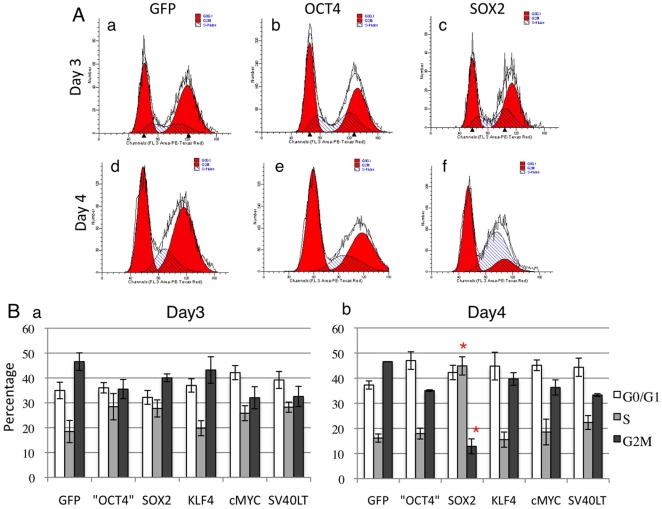
SOX2 reduced fibroblast proliferation. (A) Histogram of PI staining. The mRNA transfected were as indicated. First red peak, G0/G1 phase. Second red peak, G2/M phase. White and blue area, S phase. (B) Bar graph presentation of cell cycle profile after each mRNA transfection. The genes were indicated at the bottom. There was significantly higher percentage of S phase cells in SOX2 group on day 4 (red star).

As the expression level of individual reprogramming factors has been shown to be important for efficient reprogramming [17], we next tested the effect of each factor on cell growth following mRNA transfection. HFF were used in this series of experiments. Aliquots of 10^5^ fibroblast cells were individually microprorated with 5 µg of GFP, OCT4, SOX2, KLF4, cMYC or SV40LT mRNAs, after which the cells were seeded into 6-well plates, and cell numbers were counted three and four days later. SV40LT was chosen because it has been shown to improve the efficiency and pace of reprogramming[2], [18], although it may also increase the risk of tumorigenecity due to its ability to inhibit p53 function [19], [20], [21], [22], [23], [24], [25]. Among all the factors, we noticed that SOX2 significantly reduced cell proliferation: by day 3, OCT4, KLF4, cMYC and SV40LT transfected fibroblasts grew to 60–80% confluence, while SOX2 transfected cells were markedly less confluent (Fig. 4A–c). Cell counts showed that OCT4, KLF4, cMYC and SV40LT groups all had more than 35,000 cells, while there were only approximately 5,000 cells in SOX2 group (Fig. 4B). The GFP group had approximately 10,000 cells, indicating over-expression of GFP also had a detrimental effect on HFF fibroblast cell growth (Fig. 4B). On day 4, most groups reached 90% confluency except GFP and SOX2. We also analyzed the cell cycle profile by propidium iodide (PI) staining on day 3 and day 4. The SOX2 group had 43% of cells in S-phase on day 4, significantly higher than cells from other groups (Fig. 5A–e and B–b). This may be due to active cell cycle progression after SOX2 protein diminished from day 3. Although SOX2 showed a negative impact on cell cycle progression, as it is the co-factor for OCT4, substantial reduction of SOX2 mRNA would not be favorable for reprogramming, thus in our experiments, we used the following amounts of mRNA: OCT4∶SOX2∶KLF4∶cMYC∶SV40LT (OSKMT)  = 18∶9∶6∶6∶3 (µg/million cells), The KLF4 and cMYC mRNAs were one third of OCT4 mRNA, as this appeared to be the optimal ratio for reprogramming [17].

### Expression reprogramming factors by mRNA transfection can activate pluripotency marker genes

**Figure 6:**
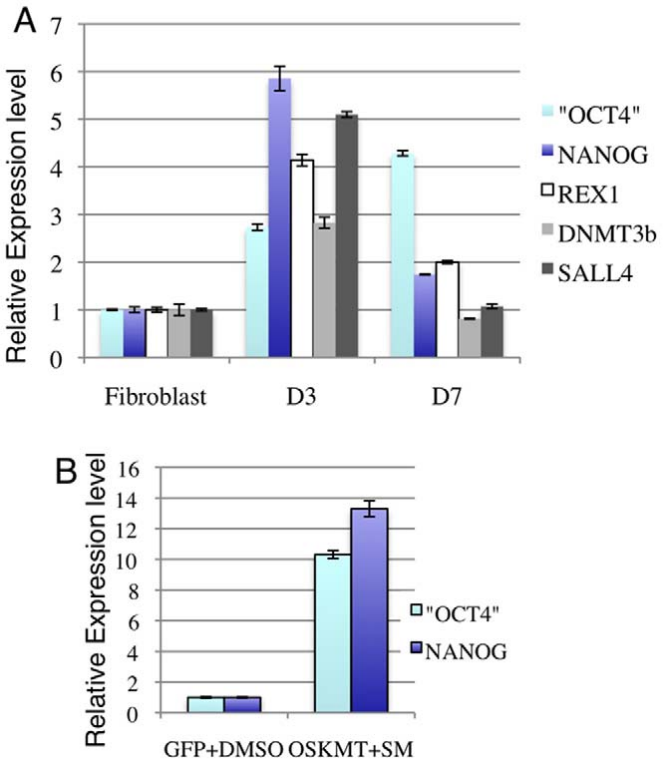
Activation of embryonic stem cell specific genes by mRNA transfection and small molecule treatment. (A) Relative expression level of ES cell specific gene (as noted) 3 and 7 days post mRNA transfection. The expression levels of these genes in fibroblast cells transfected with GFP mRNA were considered as 1. All the genes were normalized against the GAPDH level.

During somatic nuclear transfer and cell fusion experiments, pluripotency genes OCT4 and NANOG can be activated within 2–3 days [26], [27]. To test whether transient expression of reprogramming factors can activate ES cell specific genes, we microporated OSKMT mRNAs into HuF1 fibroblasts and performed quantitative RT-PCR, three and seven days after mRNA transfection in HuF1. Reverse primers specific for the endogenous OCT4 3′UTR were used to distinguish endogenous OCT4 from ectopic OCT4 mRNA. Three days after transfection, we detected significant up-regulation of ES cell marker genes OCT4, NANOG, REX1, DNMT3β and SALL4, with OCT4 increased more than two fold and NANOG more than five fold (Fig. 6A). On day seven, we detected further elevation in endogenous OCT4 level. On the other hand, the levels of NANOG, REX1, DNMT3β and SALL4 decreased compared to day three (Fig. 6A).

It has been shown that the HDAC inhibitor valproic acid, the histone methyltransferase inhibitor BIX01294 and the DNA methyltransferase inhibitor 5′-azacytidine (5′-AzaC) significantly increased the efficiency of iPS cells generation in viral transduction methods [28], [29], [30]. In our preliminary test, 5′-AzaC showed significant cytotoxicity to human fibroblast cells possibly due to inhibition of protein synthesis [31]. Therefore, we used 5′-aza-2′-deoxycytidine that causes DNA demethylation more specifically [32]. We next examined whether combining these small molecules with mRNAs of reprogramming factors could enhance the activation of pluripotency-associated genes. HuF1 cells were first microporated with the mRNA cocktail, 24 hours later, the medium was changed to human ES cell medium supplemented with valproic acid (200 µM), BIX01294 (1 µM) and 5-aza-2′-deoxycytidine (0.5 µM). After another 48 hours, cells were lysed and subjected to RT-PCR analysis. Indeed, a more than 10 fold increase in endogenous OCT4 and NANOG transcripts were detected in OSKMT mRNA plus small molecules (SM) treated cells compared with cells transfected with GFP and treated with DMSO only (Fig. 6B). These results indicate that these chemicals that influence chromatin structure can enhance the effects of short-term expression of key reprogramming factors in activating expression of the endogenous pluripotency associated transcription factor network.

**Figure 7:**
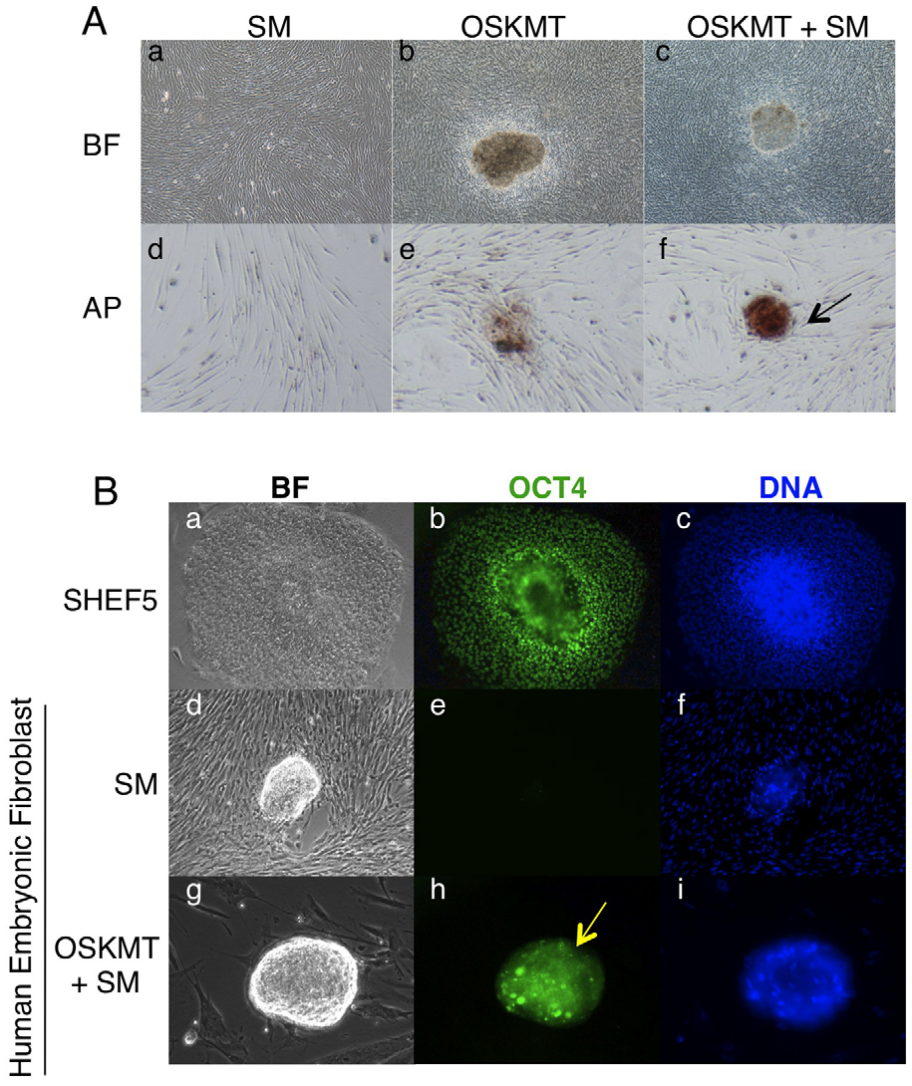
Expression of ES markers in mRNA reprogrammed cells. (A) AP positive colonies from OSKMT or OSKMT and SM treated fibroblast cells. Arrow pointing to an ES cell like colony with strong AP activity (f). (B). OSKMT and SM treated fibroblast cells expressed OCT4 (h. arrow) while small molecule (SM) treated cells did not (e). SHEF5 human ES cells were used as positive control. OCT4 in green and DNA in blue.

Complete reprogramming of human fibroblast cells to pluripotent stem cells usually takes three to four weeks [33], [34]. To test whether transient expression of OSKMT plus treatment with DNA and chromatin modifying compounds can lead to stable embryonic stem cell-like transformation after long-term culture, MRC5 human fibroblast cells were first microporated with the mRNA cocktail. Twenty four hours after microporation, the medium was changed to MEF conditioned HES medium containing BIX01294, valproic acid, and 5-aza-2′-deoxycytidine, for a further 48 hours. Afterwards, cells were cultured in MEF conditioned HES medium without any chemical compounds until confluent, approximately two weeks. Then they were passaged onto gelatin coated flasks and fed with MEF conditioned human ES medium. Some small cell aggregates became visible three weeks after microporation. However, these aggregates grew very slowly and could not be passaged. Some of the aggregates in the OSKMT plus small molecule treatment group were strongly positive for alkaline phosphatase, a marker for the ES/iPS cell (Figure 7A–f), whereas only faintly AP positive colonies were present in OSKMT mRNA treated group (Figure 7A–e). A small number of the aggregates in the OSKMT plus small molecule group could also be stained for OCT4 protein expression (Figure 7B–h), whereas none of the aggregates from small molecule or OSKMT treatment groups alone expressed OCT4. Multiple rounds of mRNA transfection and small molecule treatment were attempted, but in all instances very few cells survived, and typically senesced within a week after treatment.

### Reprogramming by mRNA and Small Molecules Caused Cell Cycle Arrest

**Figure 8:**
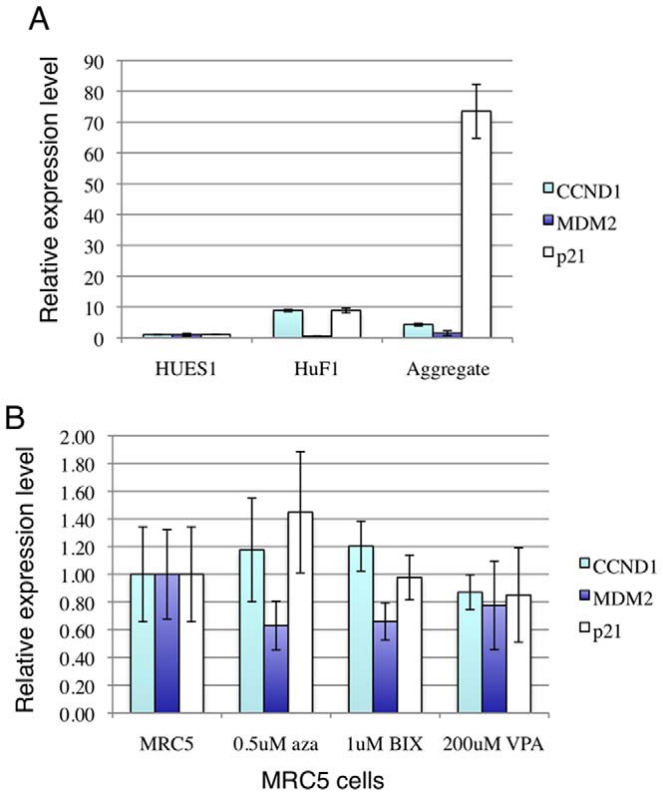
Q-PCR analysis of cell cycle and DNA damage response genes. All values were normalized against GAPDH. (A) p21 RNA was markedly up-regulated in cell aggregates. The expression values in HUES1 ES cells were set as “1”. (B) The expression levels of CCND1, MDM2 and p21 were not significantly altered by any of the small molecules. The expression values in DMSO treated MRC5 cells were set as “1”.

Induction of pluripotency in somatic cells needs to overcome the barrier imposed by DNA damage repair machinery [19], [20], [21], [22], [23], [24], [25]. The reason that the apparently reprogrammed cells that we observed in our experiments failed to proliferate could have been due to cell cycle arrest following a DNA damage response. To test this, several cell aggregates in OSKMT plus small molecule group were manually picked 4 weeks after transfection and subjected to RT-PCR analysis. p21, a target of p53 and an inhibitor of cell cycle progression, was significantly up-regulated in cell aggregates compared with in HUES1 ES cells and fibroblast cells transfected with GFP (Fig. 8A), while 48 hrs treatment with any of the small molecules did not lead to any substantial change in these genes (Fig. 8B) This suggests that while transient OSKMT expression combined with small molecule treatment induced a certain degree of reprogramming, it also triggered DNA damage response and cell cycle arrest.

## Discussion

In this study, we have developed a protocol to efficiently introduce mRNAs encoding OCT4, SOX2, KLF4, cMYC and LT into human fibroblast cells. Moreover, we showed that the level of protein expression tightly correlates with the amount of input mRNA. mRNA exhibited much higher transfection efficiency and less cytotoxicity than DNA. We observed less cell death in fibroblast cells transfected with 40 µg of mRNA than 3 µg of plasmid DNA (data not shown). Over expression of OCT4 promoted cell proliferation while SOX2 alone seemed to have opposite effect. In the light of the cell cycle studies, we modified the ratio of mRNAs to reduce the negative impact of SOX2. Our results suggest that it is possible to combine mRNA factors of different concentrations to create an optimized reprogramming mix to improve reprogramming efficiency.

Transfection of mRNA encoding five reprogramming factors can activate normally silenced embryonic genes within a few days. This result is in agreement with several recently published studies. In cell fusion experiments, pluripotency genes began to express within one to two days following somatic cells fusion with ES cells even in the absence of SOX2 [35], [36], [37], [38]. OCT4 and NANOG demethylation occurred just one day after fusion [27]. When a somatic nucleus was place into a mouse zygote, the OCT4 gene was activated after only two cell cycles [26]. These reports together with ours suggest that given sufficient amount of reprogramming factors, the de-differentiation process can be initiated rather rapidly. This therefore raises the question of why iPS cells can only be obtained after stable expression of defined factors for 3–4 weeks, while an enucleated oocyte can reprogram somatic nucleus within a few days [39]? In addition to OCT4, SOX2, KLF4 and cMYC, enucleated oocytes contain many regulators of chromatin modification, cell cycle and DNA damage response [40], which may be responsible for their robust reprogramming ability. Indeed, oocyte factors such as activation-induced cytidine deaminase (AID) and histone demethylase Jhdm2a can demethylate DNA and histones respectively and are required for reprogramming through the ES cell fusion method [27], [35]. It is possible that by adding the proper amount of “helper” factors to the Yamanaka 4-factor or Thomson 4-factor [33], [34], the reprogramming process can be significantly accelerated.

Small molecules that can erase somatic chromatin and DNA modifications have been shown to greatly improve the reprogramming efficiency from mouse fibroblast cells [28], [30]. The small molecule BIX-01294, an inhibitor of the G9a histone methyltransferase, when combined with OCT4 and KLF4, reprogrammed neural stem cells more efficiently than using OCT4, KLF4 and cMYC [30]. While HDAC inhibitor valproic acid, improves reprogramming efficiency by more than 100-fold [28]. We found that brief treatment with 5-aza-2′-deoxycytidine, BIX-01294 and valproic acid following mRNA transfection further increased the activation of pluripotency genes than mRNA transfection alone. However, during our attempt of multiple rounds of microporation transfection, such treatment caused massive cell death. Although colonies positive for AP and OCT4 appeared following just one round of mRNA transfection and small molecule treatment, these cells still activated high levels of p21 and failed to expand. Thus, additional chemical compounds that support cell survival or relieve DNA damage response will be beneficial for generating iPS cells using mRNA and small molecule approach. Caution should also be taken while using these reagents. For example, SV40LT is known to inhibit tumor suppressor p53 function and cause cancer-like cellular transformation [41]. Use of genes or compounds to inhibit DNA damage in order to facilitate reprogramming may increase the risk of tumorigenicity of resulting iPS cells.

Recently, there were two reports on using mRNA generate iPS cells. Yakubov and colleagues obtained similar AP positive colonies as us, however, no differentiation analysis were done, thus it is hard to evaluate the pluripotency of their iPS cells [42]. Angel and Yanik found that long RNA transfection activated innate immunity that caused significant cell death [43]. Their result is in agreement with our observation that repeated mRNA transfection resulted in cell growth arrest and death.

In summary, our results demonstrate that by optimizing the combination and dosage of mRNA and small molecules, it is possible to reprogram cell fate without using any DNA. This strategy could be exploited to generate cells with therapeutic values. Recently, it was shown that fibroblast cells can be reprogrammed to neurons by defined factors within 3–5 days [7]. Adenovirus transduction of NGN3, PDX1 and MAFA in adult pancreas led to appearance of new insulin secreting cells after 3 days, indicating trans-differentiation from exocrine β cells to endocrine is a relatively fast process [44]. These time windows fall into the range that can be fulfilled by mRNA transfection which is 2-3 days. While our manuscript was in revision, Warren et al successfully generated human iPS cells using mRNA [45]. The key to their success is to suppress interferon response triggered by repeated mRNA transfection. mRNA has the advantage of high transfection efficiency, controllability and avoidance of genetic modification. Once the innate immune response activated by introducing large amount of long RNAs can be overcome, it will be a very useful approach for cellular reprogramming.

## Supporting Information

Table S1 Q-PCR primers.
(0.03 MB DOC)
