# Supplementary material for: Activation of Pluripotency Genes in Human Fibroblast Cells by a Novel mRNA Based Approach
Source: PLoS One. 2010 Dec 30;5(12):e14397. doi: 10.1371/journal.pone.0014397 (PMC3012685; doi:10.1371/journal.pone.0014397)
Supplement: Table S1 — Q-PCR primers. (0.03 MB DOC) [file pone.0014397.s001.doc]

Table S1. Q-PCR primers.

| Gene | Accession | Forward | Reverse | T. |
| --- | --- | --- | --- | --- |
| CCND1 | NM_053056 | gggggcgtagcatcatagta | tgtgagctggcttcattgag | 60 |
| DNMT3β | NM_006892 | caggagacctaccctccaca | tgtctgaattcccgttctcc | 60 |
| GAPDH | NM_002046 | tgatgacatcaagaaggtggtgaag | tccttggaggccatgtgggccat | 60 |
| MDM2 | NM_002392 | gtgatcttggctcactgcaa | acgaggtcaggagatcgaga | 60 |
| NANOG | NM_024865 | ggatggtctcgatctcctga | cctcccaatcccaaacaata | 60 |
| OCT4 | NM_002701 | cgaccatctgccgctttgag | ccccctgtcccccattccta | 60 |
| p21 | NM_000389 | ttagcagcggaacaaggagt | gccgagagaaaacagtccag | 60 |
| REX1 | NM_174900 | gctgaccaccagcacactaggc | tttctggtgtcttgtctttgcccg | 60 |
| SALL4 | NM_020436 | gccgtgaagaccaatgagat | ctccttccacgcaagttctc | 60 |
